# The Coming Man Physically

**Published:** 1880-12

**Authors:** 


### THE COMING MAN PHYSICALLY.

In Prof. C. W. Emerson's lecture on the "Coming Man," delivered recently in Boston, before the Moral Educational Association, he gave this outline of his vision of the coming man physically: "We cannot hope that his physical development will be absolutely perfect, but he will be so far ahead of the present man that, could we see him in a vision, he would seem to us perfect, as indeed we ourselves would seem perfect to the people of ancient history. There must be great physical improvement in the future man, because all the hindrances of health are being taken away. We are getting interested in the well-being of our bodies, superstitions are vanishing, we have learned that pestilence and plague are the result of bad sewerage and filth, and that the remedy lies within our reach. Statistics bear out this theory that man is advancing toward physical perfection. There is greater longevity now than in the past, and men of seventy are now stronger than once men of sixty were. Physically, therefore, the coming man will be more robust. And by this is not meant more muscular, but possessing more vitality of the whole system. As man becomes more healthy, he will become less susceptible to bad habits and temptations. A perfectly well man is never criminal. It is when the nerves are deranged by drink, evil habits or tobacco (for no man is perfectly well who has used tobacco for a single month) that temptation cannot be resisted and crime follows."

---

THEY had not been married long, so they sat down to play "checkers." In the middle of the game she said:

"Then do I jump these two men and get a king? Of course I do. Crown me. I've got the first king," and she chuckled hysterically.

"No, you ain't, either. I didn't mean that move," said he. "If you can't play checkers without cackling like a hen you had better give it up. I'll take that back and move here; now, so. Now you can move."

"Over here?" asked the wife.

"Certainly. That's very good," and her husband gobbled two men.

"I didn't see that. I'd rather put it here," she remonstrated.

"Too late now," said he, pegging away for the king row. "You should study your moves first."

